# Chemosensitization of HT29 and HT29-5FU Cell Lines by a Combination of a Multi-Tyrosine Kinase Inhibitor and 5FU Downregulates ABCC1 and Inhibits PIK3CA in Light of Their Importance in Saudi Colorectal Cancer

**DOI:** 10.3390/molecules26020334

**Published:** 2021-01-11

**Authors:** Ashraf N. Abdalla, Waleed H. Malki, Amal Qattan, Imran Shahid, Mohammad Akbar Hossain, Muhammad Ahmed

**Affiliations:** 1 Department of Pharmacology and Toxicology, College of Pharmacy, Umm Al-Qura University, Makkah 21955, Saudi Arabia; whmalki@uqu.edu.sa (W.H.M.); iyshahid@uqu.edu.sa (I.S.); mahossain@uqu.edu.sa (M.A.H.); mmsiddiqui@uqu.edu.sa (M.A.); 2 Medicinal and Aromatic Plants Research Institute, National Center for Research, Khartoum 2424, Sudan; 3 Breast Cancer Research, Department of Molecular Oncology, King Faisal Specialist Hospital and Research Centre, Riyadh 11211, Saudi Arabia; akattan@kfshrc.edu.sa; 4 Department of Biochemistry and Molecular Medicine, School of Medicine and Health Sciences (SMHS), George Washington University, Washington, DC 20073, USA; 5 Department of Pharmacology and Toxicology, Faculty of Medicine, Umm Al-Qura University, Makkah 21955, Saudi Arabia

**Keywords:** Saudi colorectal cancer, kinase pathway profiling, PIK3CA, 5FU, drug resistance, ABCC1

## Abstract

Colorectal cancer (CRC) remains one of the main causes of death worldwide and in Saudi Arabia. The toxicity and the development of resistance against 5 fluorouracil 5FU pose increasing therapeutic difficulties, which necessitates the development of personalized drugs and drug combinations. Objectives: First, to determine the most important kinases and kinase pathways, and the amount of ABC transporters and KRAS in samples taken from Saudi CRC patients. Second, to investigate the chemosensitizing effect of LY294002 and HAA_2020_ and their combinations with 5FU on HT29, HT29-5FU, HCT116, and HCT116-5FU CRC cells, their effect on the three ABC transporters, cell cycle, and apoptosis, in light of the important kinase pathways resulting from the first part of this study. Methods: The PamChip^®^ peptide micro-array profiling was used to determine the level of kinase and targets in the Saudi CRC samples. Next, RT-PCR, MTT cytotoxicity, Western blotting, perturbation of cell cycle, annexin V, and immunofluorescence assays were used to investigate the effect on CRC, MRC5, and HUVEC cells. Results: The kinase activity profiling highlighted the importance of the PI3K/AKT, MAPK, and the growth factors pathways in the Saudi CRC samples. PIK3CA was the most overexpressed, and it was associated with increased level of mutated KRAS and the three ABC transporters, especially ABCC1 in the Saudi samples. Next, combining HAA_2020_ with 5FU exhibited the best synergistic and resistance-reversal effect in the four CRC cells, and the highest selectivity indices compared to MRC5 and HUVEC normal cells. Additionally, HAA_2020_ with 5FU exerted significant inhibition of ABCC1 in the four CRC cells, and inhibition of PIK3CA/AKT/MAPK7/ERK in HT29 and HT29-5FU cells. The combination also inhibited EGFR, increased the preG_1_/S cell cycle phases, apoptosis, and caspase 8 in HT29 cells, while it increased the G_1_ phase, p21/p27, and apoptosis in HT29-5FU cells. Conclusion: We have combined the PamChip kinase profiling of Saudi CRC samples with in vitro drug combination studies in four CRC cells, highlighting the importance of targeting PIK3CA and ABCC1 for Saudi CRC patients, especially given that the overexpression of PIK3CA mutations was previously linked with the lack of activity for the anti-EGFRs as first line treatment for CRC patients. The combination of HAA_2020_ and 5FU has selectively sensitized the four CRC cells to 5FU and could be further studied.

## 1. Introduction

Colorectal cancer (CRC) ranks third and fourth amongst the deadliest and diagnosed cancers worldwide, respectively [1]. In Saudi Arabia, it occupies the first and third top occurring malignancy in both sexes, respectively [2]. In CRC patients, the binding of VEGF/VEGFR results in migration, invasion, and survival of cancer cells, while the binding of EGF/EGFR results in cell proliferation, vascular permeability, and cancer cell survival, all leading to angiogenesis which is one of the main derivers of CRC [3]. While surgery is the first intervention for CRC patients, metastasis occurring in at least a quarter of the patients necessitates the use of suitable drugs [1]. 5FU is one of the oldest and efficient first-line CRC drugs, which is categorized in the conventional chemotherapeutic group [4]. It disturbs the metabolism of nucleosides leading to cell death, but its efficiency is marred by resistance of CRC cells to 5FU [5]. However, considering specific CRC predictive biomarkers, the resistance to 5FU and many other CRC drugs can be overcomed by the use of targeted therapies including bevacizumab as the first-line anti-vascular endothelial growth factor-A (anti-VEGF-A) drug. One of the advantages of the anti-VEGFR (anti-angiogenic) drugs is that they can be prescribed for wider groups of CRC patients as they have more diverse biomarkers and predictive factors of response compared to the anti-EGFR drugs [6,7]. As an example, the overexpression of PIK3CA is one of the important predictive biomarkers for the use of anti-VEGFR drugs, but it is not the case for the anti-EGFR drugs [8,9]. Nevertheless, most of the anti-angiogenic drugs are hindered by several toxicities including gastro-intestinal (GIT) disturbances, high blood pressure, nephrotoxicity, and impaired wound healing, all which decrease the clinical overall survival in CRC patients [3,10,11,12].

The tyrosine kinases identified from the arrays and sequencing-based transcriptomic studies, considered important targets and predictive biomarkers of CRC, were the most common mutated kinases KRAS, RAS/RAF/MAPK, PIK3CA/AKT, TP53, SMAD4, Src-NOS, PLC/ERK, PDGFR/ERK and APC [3,7,10,13]. Previous immunohistochemistry and tissue microarray studies showed that the following mutations are overrepresented in Saudi CRC patients compared with Caucasian CRC patients: telomerase reverse transcriptase (hTERT) in elders, PIK3CA in all ages (exon 9, 12% higher in Saudis), and Rad3-related protein (ATR) in Saudi females compared to males [14]. Moreover, many other CRC related genes and alterations were reported in Saudi CRC patients using cytogenetic studies including NARS, ATP5A1, CTCFL, and PARP-1 [15,16]. HER2 and TOP2A, which are important biomarkers of breast cancer, were also detected in Saudi CRC patients but in lower amplifications compared to Saudi breast cancer patients [17].

Most CRC patients experience resistance to drugs during their treatment course. This is due to many mechanisms, mainly the overexpression of the ATP-binding cassette transporters (ATP), namely the ABCB1, ABCC1, and ABCG2 transporters which efflux drugs out of the cancer cells [18]. The upregulation of Bcl-2 and EGFR, increase of the PI3K/AKT, and downregulation of p53/Bax are all important cellular mechanisms which are strongly correlated to the upregulation of the ABC transporters ending with evasion of apoptosis and drug resistance [18]. The overexpression of ABCB1 and MRP in CRC patients was previously associated with resistance to methotrexate, camptothecins, hydroxyurea, and 5FU treatment regimens [19,20]. Additionally, the resistance to VEGFR inhibitors was attributed to acquired receptor mutations in PIK3CA/AKT, ERK, HER2, or EGFR [11]. In a previous study, the three transporters were found to be highly expressed in the blood of Saudi CRC patients [21]. While other studies showed the association between the overexpression of ABCB1 and ABCG2, and the treatment outcomes in Saudi CRC patients [22]. At the in vitro level, many studies have reported variable overexpression amounts of the ABCs in HT29 colorectal cancer cells [23,24,25,26].

Due to the overlap and complexity of the angiogenesis pathways which results in poor CRC treatment outcomes, toxicities, and drug resistance, there is growing need for the development of personalized medicines that are more ethnicity relevant and effective [7,27]. The identification of differentially enriched gene expressions and key pathways are important backbones of precise drug discovery. A number of high-tech platforms are used for these identifications [28] including the PamChip^®^ peptide micro-array-based kinase activity profiling, which is used to study the kinase activity in cell lysates and clinical samples. It enables the determination and assessment of the kinase’s levels, compared to genetic sequencing which can only identify mutations in kinases in the human genome. The STK (serine/threonine kinase) and the PTK (phosphorylation by tyrosine kinase of immobilized peptides) are the two assays that can be utilized in the PamChip^®^ platform. In the STK, a combination of FITC-labeled secondary antibody and anti-phospho-serine/threonine antibodies can be used for detection of peptide phosphorylation, while the FITC-labeled and anti-phospho-tyrosine antibodies are used in the PTK application [29,30,31,32]. Thus, the determination of the differentially overexpressed and CRC-associated kinases in a specific population could facilitate better selection of CRC drugs or drug combinations that can reverse resistance.

The resistance to 5FU and other drugs can also be reversed by the use of combination strategies yielding up to 50% improvement of the response rates [5]. In a phase II clinical study, the clinical response and safety of 5FU were improved after its combination with oxaliplatin and irinotecan to 24–74% [3]. Chemosensitization of CRC cells to the cytotoxic effect of 5FU can be induced by many agents through inhibiting the PI3K/AKT and CDK4, and increasing cell numbers in the preG_1_, G_1_, and S phases leading to apoptosis [5].

**Figure 1:**
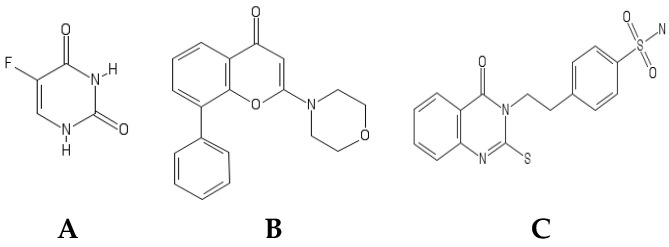
Molecular structures of (**A**) 5FU, (**B**) LY294002, and (**C**) HAA_2020._

Many members of the drug class quinazoline were reported to target tyrosine kinases as the case of cediranib (VEGFR/PDGFR), sunitinib malate (VEGFR/PDGFR/c-KIT/FLT-3), and the anti-CRC drug vatalinib (VEGFR-1/VEGFR-2). Notably, four out of the six FDA-approved quinazolines are EGFR inhibitors: gefitinib, erlotinib, afatinib, and lapatinib [33,34]. It was also reported that the EGFR and HER2/3 inhibitor sapitinib, which contains a quinazoline moiety, showed chemosensitizing effect on resistant CRC cells by downregulation of the ABCB1 transporter [35]. A previously synthetized novel quinazoline derivative [36] will be used in this study (named HAA_2020_, Figure 1C). HAA_2020_ previously exhibited multi-tyrosine kinase inhibitory effects against VEGFR-2, EGFR, and Her2, and it also showed cytotoxic activity against HT29, MCF7, HL60, and K562 cells [36].

The aim of the first part of this study was to explore the significant targetable kinases and kinase pathways in samples taken from Saudi CRC patients using the PamChip^®^ peptide microarray, and to assess the amount of KRAS and the three ABC transporters in the lysates of the same patient samples. In the next step, LY294002 and HAA_2020_ were tested alone and in combination with 5FU to investigate their possible chemosensitizing effect on HT29, HT29-5FU, HCT116, HCT116-5FU, MRC5, and HUVEC cell lines as in vitro model, and to test their effect on the important kinase pathways resulting from the Saudi CRC samples, the ABC transporters, in addition to their effect on cell cycle and apoptosis.

## 2. Results

### 2.1. Criteria of the Saudi CRC Patients

Patients were selected randomly for this study, and most of their ages were between 50–59 years, while the other patients were distributed over all age groups. Most of the patients were females (70%), compared to 30% males. The body mass index (BMI) of 90% of patient’s ≥ 25. Further characteristics of the CRC samples are shown in Table 1.

### 2.2. Tyrosine and Serine/Threonine Activities in the CRC Samples

To our knowledge, this is the first report of using the PamChip^®^ peptide microarrays to determine the tyrosine and serine/threonine kinase activities in Saudi CRC samples. The resulting data were analyzed and deposited in the Metacore, where the identities of the significantly phosphorylated proteins were matched in the functional ontologies in MetaCore with gene identities. The *p*-value represents the random intersection probability between the identities and targets (lower *p*-value represents higher entity, dataset relevancy).

#### 2.2.1. Enrichment Pathway Analysis

**Figure 2:**
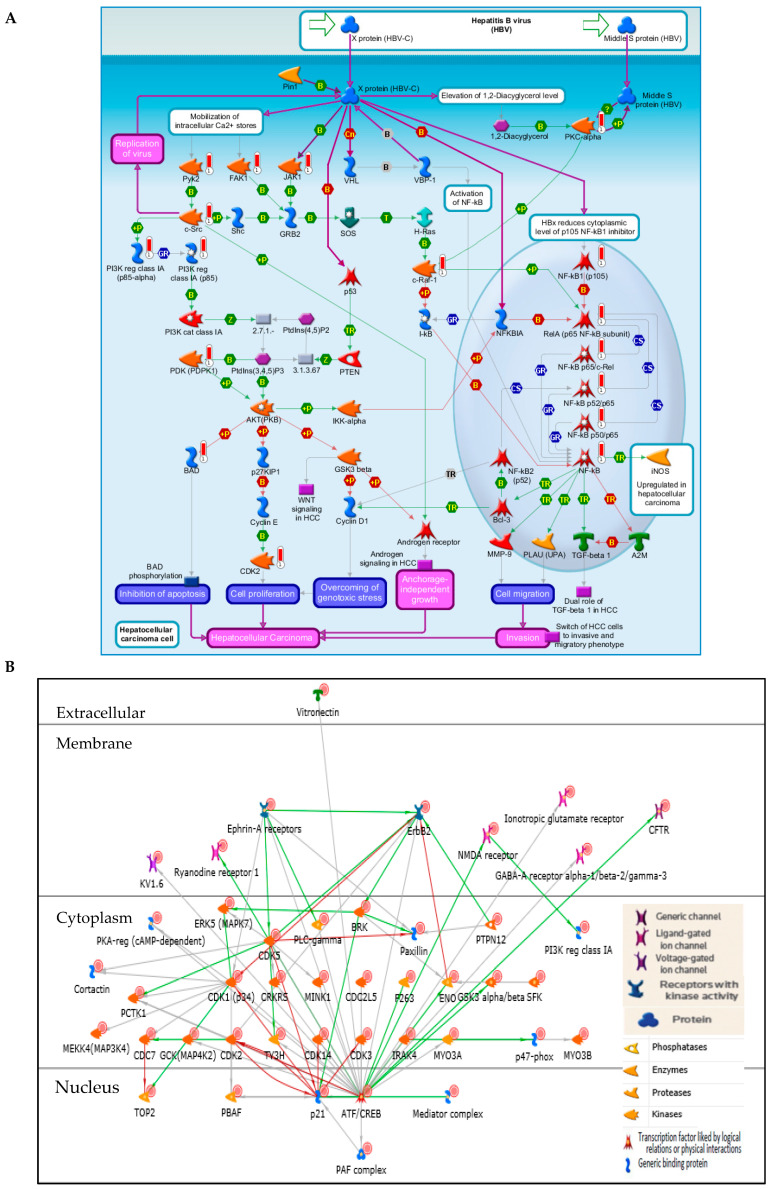
(**A**) Pathway map of PIK3CA/AKT. Symbols looking like a thermometer represent the kinase analysis data. Red color represents protein levels of phosphorylation. (**B**) Peptides with the highest signals in the PI3K/AKT network (PIK3CA red-circled).

The Metacore enrichment pathway analysis of the Saudi CRC samples showed the top ten important pathways. The PI3K/AKT pathways ranks the first, with the lowest log *p*-value (Table 2, Figure 2A,B), followed by the inhibition of Ephrin receptors in colorectal cancer (Supplementary material, Figure S1A), growth factor driven development (Supplementary material, Figure S1B), and the oxidative stress ROS-mediated MAPK activation (Supplementary material, Figure S1C) in the fourth rank. Notably, the development via EGFR, and VEGF signaling via VEGFR2 pathways ranked six and seven. The PI3K regulatory subunit class IA (PIK3CA) was overexpressed in eight out of the top ten enrichment pathway analyses (Table 2). Additionally, the cyclin dependent kinases (CDK 1, 2, 3, 5, and 14) and p21 (cyclin dependent kinase inhibitor, which regulates G_1_ and S cell cycle phases) showed high signals (Figure 2B).

#### 2.2.2. Network and GO Processes

**Figure 3:**
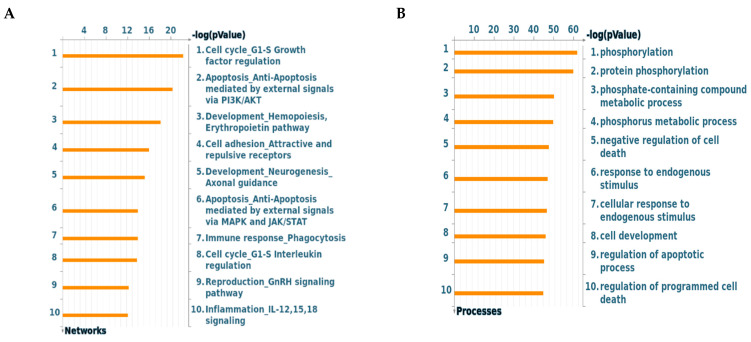
(**A**) The highest-ranking networks in the Saudi CRC samples (Supplementary material S1: process network). (**B**) Gene ontology (GO) processes of the Saudi CRC samples, sorted by statistically significant processes (Supplementary material S1: GO processes).

The network processes of the Saudi CRC samples are shown by the network of protein interactions (Figure 3A). The cell cycle G_1_/S growth factor regulation tops the list, followed by apoptosis and anti-apoptosis mediated by PI3K/AKT and MAPK-JAK/STAT signaling pathways. Furthermore, the top detected gene ontology (GO) processes were phosphorylation and protein phosphorylation. The regulation of apoptosis appeared twice among the top ten processes (Figure 3B).

### 2.3. Real-Time PCR of the CRC Pateints’ Samples

**Figure 4:**
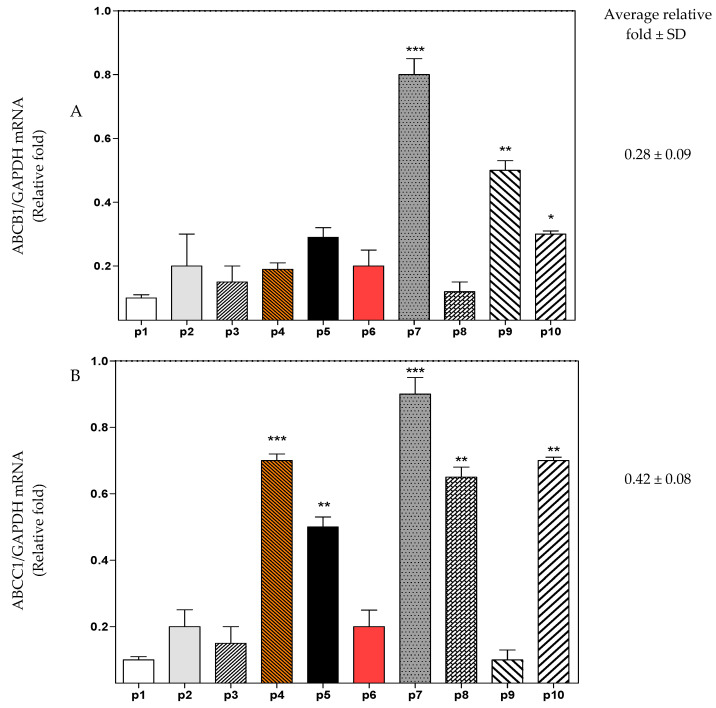

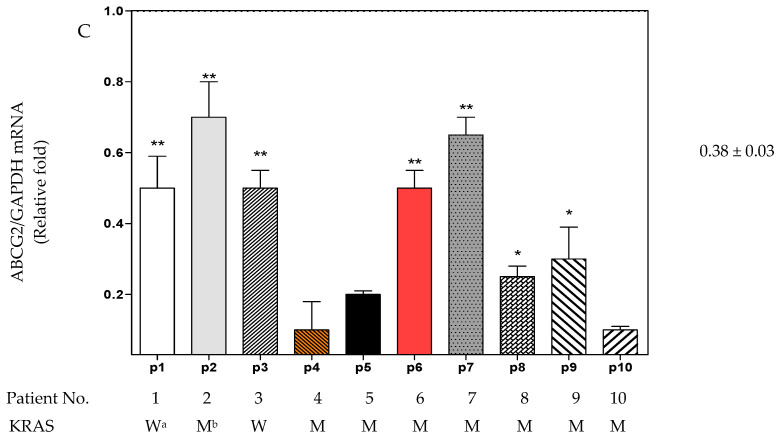
Total mRNA was isolated from the 10 Saudi patients (p1–p10) and quantified by RT-PCR with (**A**) ABCB1, (**B**) ABCC1, and (**C**) ABCG2 primers. The results were expressed as relative fold change (average ± SD, *n* = 3, x2 independent experiments) compared with GAPDH (1-fold change). W ^a^: wild type KRAS, M ^b^: mutated KRAS. Statistical differences (one-way ANOVA and Tukey’s post-hoc): *p* < 0.05 (*), *p* < 0.01 (**), *p* < 0.001 (***) were considered significant.

Following immediate RNA extraction of samples taken from–80 °C, the mRNA amount of ABCB1, ABCC1, and ABCG2 were investigated by RT-PCR in each of the frozen Saudi CRC samples. The level of mRNA of the three transporters was compared to GAPDH. The result showed variability among the different samples within each transporter. Comparing the average relative fold change of each transporter, the amount of detected ABCC1 was the highest followed by ABCG2 and ABCB1 (0.42, 0.38, and 0.28 respectively, Figure 4). Patients No. 7, 8, 9, and 10 showed the most significant amounts of the three transporters. The KRAS status of each patient was detected by RT-PCR and was tabulated at the end of Figure 4 either as wild type KRAS or mutated KRAS. Patients No. 1 and 3, who have the lowest amount of the ABC transporter genes, showed no KRAS mutation.

### 2.4. Combination Cytotoxicity and Selectivity Studies

The kinase-based pathway analysis showed the importance of PI3K/AKT, MAPK, and EGFR/VEGF signaling in the tumorigenesis of the ten Saudi CRC samples. Thus, this result was used for the selection of previously described suitable compounds to explore their combinatory effect with 5FU. For inhibition of the PI3K/AKT, the LY294002 was selected. Additionally, the novel quinazoline derivative (HAA_2020_) was selected because of its previously shown potent EGFR, VEGFR-2, and Her2 inhibition activities [36]. The MTT cytotoxicity assay of 5FU, LY294002, HAA_2020_, and their combinations (72 h) was performed in HT29, HT29-5FU, HCT116, and HCT116-5FU cells (IC_50_ shown in Table 3 and Table 4). In HT29 and HCT116 cells, 5FU was the most potent, followed by HAA_2020_ and LY294002. Next, each of the two drugs or both were combined with 5FU at one fixed ratio according to their IC_50_ (1:1 or 1:1:1, respectively). Combining HAA_2020_ with 5FU exerted the best cytotoxicity and CI, whereas the combinations including LY294002 exerted the lowest cytotoxicity and highest CI in both cells. HT29 cell line was more sensitive for the different treatments compared to HCT116.

In the next step, HT29-5FU and HCT116-5FU cells showed resistance to 5FU (231– 300 fold increase in its IC_50_ value compared to its activity in the HT29 and HCT116 cells), whereas LY294002 and HAA_2020_ only lost 2.42–3.5 folds compared to their activity in the parent HT29 and HCT116 cells. Among the four combinations, only HAA_2020_ combined with 5FU showed synergistic activity in HT29-5FU and HCT116-5FU cells (CI: 0.80 and 0.95, respectively, Table 3 and Table 4), along with the highest fold reversal activity (FR) in the two cell lines (7.5 and 5.5, respectively).

In general, the toxicity of the CRC drugs plays a major drawback in their safety and efficacy [3]. Thus, the MRC5 and HUVEC cells were selected for determination of the selectivity of the three agents and their combinations. The selectivity index (SI) of 5FU in HT29 and HCT116 cells compared to MRC5 was high (134.4 and 162.6, respectively), and it was much less for HAA_2020_ and LY294002 (Table 5). Combining HAA_2020_ to 5FU significantly improved the selectivity of both compounds to 68.6–206 in the two cells compared to MRC5. While the involvement of LY294002 decreased the selectivity, either when combined with each of 5FU or HAA_2020_, or with both. The selectivity of all single and combined treatments in either HT29-5FU or HCT116-5FU cells compared to MRC5 cells was below 1, except for HAA_2020_ or HAA_2020_ combined with 5FU, which was 1.1–2.1 (Table 5). Testing the selectivity of the single and combined treatments in the four cell lines compared to HUVEC (Table 6), showed that the combination of HAA_2020_ and 5FU exhibited the highest selectivity in HT29 and HCT116 cells (541 and 180.6, respectively), and also in HT29-5FU and HCT116-5FU cells (3.0 and 3.4, respectively). Thus, it was shown that while the combination of HAA_2020_ and 5FU sensitized each of the four cell lines, it exerted the best combination indices and resistance reversal effects in HT29 and HT29-5FU cells, compared to HCT116 and HCT116-5FU cells.

### 2.5. Real-Time PCR of ABC Transporters in HT29, HCT116, HT-5FU and HCT116-5FU Cells

**Figure 5:**
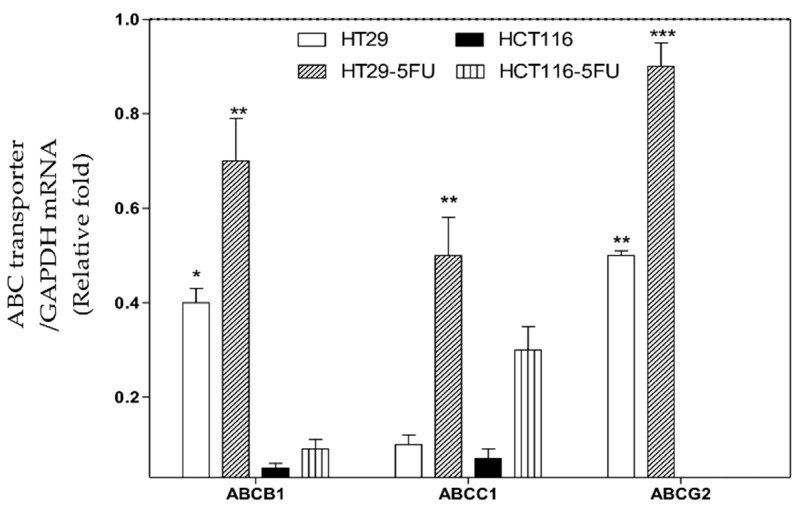
Total mRNA was isolated from HT29, HT29-5FU, HCT116, and HCT116-5FU cells and quantified by RT-PCR with ABCB1, ABCC1, and ABCG2 primers. The results were expressed as relative fold change (average ± SD, *n* = 3, ×2 independent experiments) compared with GAPDH (1-fold change). Statistical differences (one-way ANOVA, Tukey’s post-hoc): *p* < 0.05 (*), *p* < 0.01 (**), and *p* < 0.001 (***) were considered significant.

Previous reports in the literature about the amounts of ABC transporters in HT29, HCT116, HT-5FU, and HCT116-5FU cells are variable [23,24,25,26], thus, the RT-PCR was conducted in this study to quantify the levels of ABC transporters expression in the four cells. Figure 5 shows that the ABC transporters were more expressed in the HT29-5FU cells compared to the other cells. ABCG2 was the highest in its expression in HT29 and HT29-5FU cell lines. HCT116 and HCT116-5FU cells showed less expression of ABCB1 and ABCC1 compared to HT29 and HT-5FU, while they did not show ABCG2.

**Figure 6:**
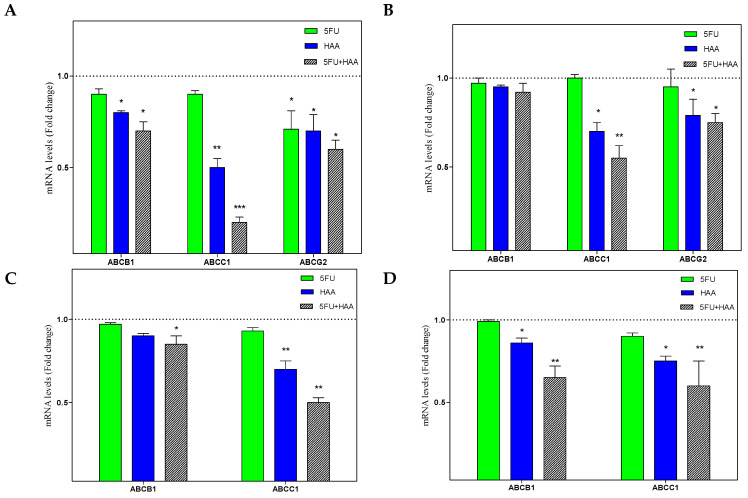
The inhibitory effect (72 h) of vehicle control, 5FU (0.25 µM), HAA_2020_ (3 µM), or their combination on the expression of mRNA of ABCB1, ABCC1, and ABCG2 in (**A**) HT29, (**B**) HT29-5FU, (**C**) HCT116, and (**D**) HCT116-5FU cells was quantified by RT-PCR. The data represent the mean ± SD of the fold change related to vehicle control (fold change = 1 dashed line, *n* = 2, ×2 independent experiments). *p* < 0.05 (*), *p* < 0.01 (**), and *p* < 0.001 (***) were considered significant.

In the next step, the effect of 5FU, HAA_2020_, and their combination were tested against the four cells (Figure 6A–D). In HT29, 5FU caused a non-significant inhibitory effect on ABCB1, compared to HAA_2020_ or the combination. The same inhibitory effect of 5FU was observed on ABCC1 and ABCG2, while HAA_2020_ and the combination showed more significant inhibition on ABCC1. The three genes were less affected by 5FU, HAA_2020_, or their combination in HT29-5FU compared to HT29, whereas ABCC1 and ABCG2 were the most affected by the treatments (Figure 6A,B). Similarly, HAA_2020_ alone or combined with 5FU showed significant inhibitory effect on ABCB1 and ABCC1 in HCT116 and HCT116-5FU cells, but the latter were less sensitive to treatment effect compared to HT29 and HT29-5FU cells (Figure 6C,D). The combination of 5FU and HAA_2020_ showed better ABC transporter inhibitory effects compared to each of the two compounds alone.

### 2.6. Real-Time PCR and Western Blotting in HT29 and HT29-5FU Cells

**Figure 7:**
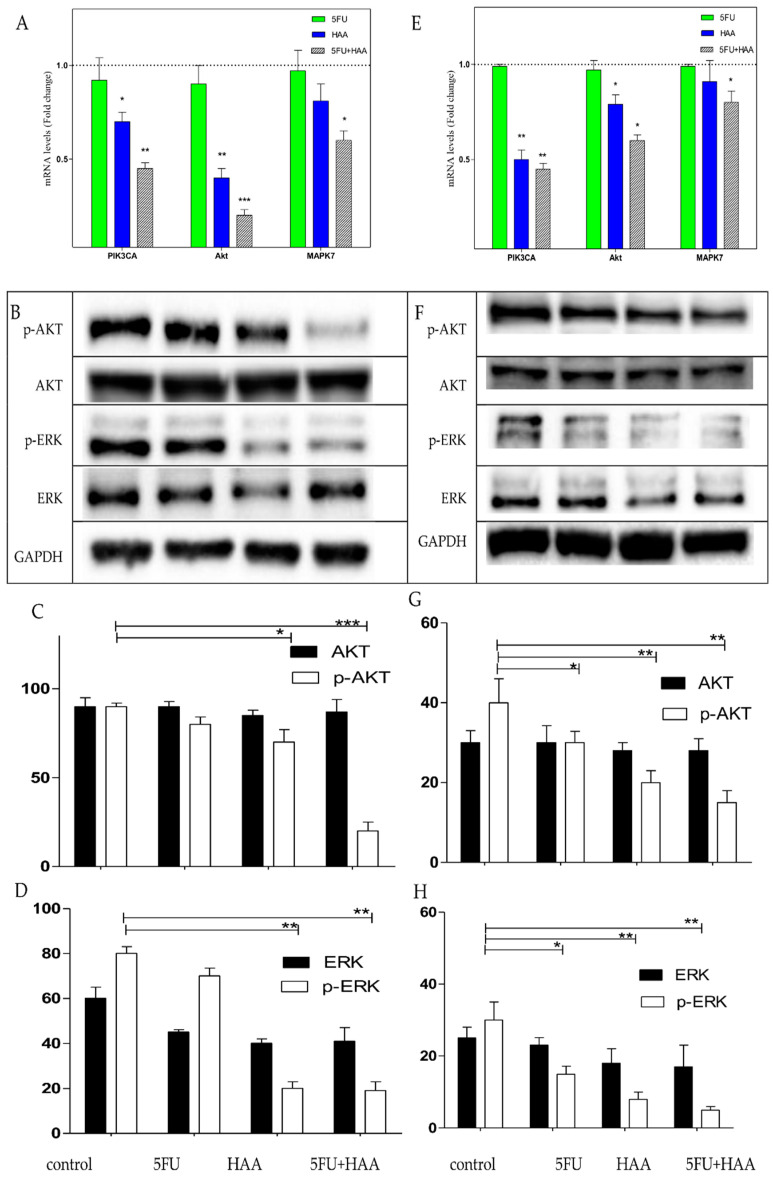
The expression of (**A**) PIK3CA, AKT, and MAPK7, and (**B**–**D**) AKT, p-AKT, ERK, and p-ERK in HT29 cells, and expression of (**E**) PIK3CA, AKT, and MAPK7, and (**F**–**H**): AKT, p-AKT, ERK, and p-ERK in HT29-5FU cells. All cells were treated with vehicle, 5FU (0.25 µM), HAA_2020_ (3 µM), and their combination for 72 h. Results in (**A**) and (**E**) represent the mean ± SD of the mRNA fold change related to vehicle control (fold change = 1 dashed line). Results in (**C**,**D**,**G**,**H**) represent the mean ± SD of the fold change percentage (%, Y axis) of the relative protein levels normalized to GAPDH, *n* = 2, ×2 independent experiments. *p* < 0.05 (*), *p* < 0.01 (**), and *p* < 0.001 (***) were considered significant.

Based on the previous results, HT29 and HT29-5FU cells were further investigated. First, the mRNA expressions of PIK3CA, AKT, and MAPK7 were investigated by RT-PCR after treatment with vehicle control, 5FU (0.25 µM), HAA_2020_ (3 µM) and their combination (*n* = 2) for 72h. In both cell lines, HAA_2020_ combined with 5FU inhibited PIK3CA, AKT, and MAPK7 more compared to each of the compounds alone_,_ but that inhibition was more significant in the HT29 cells (Figure 7A,E). In agreement with this result, the Western blotting of lysates from the two cell lines treated with 5FU (0.25 µM), HAA_2020_ (3 µM), and their combination for 72 h showed significant inhibition of phosphorelated AKT in both cells (Figure 7B,C for HT29, and Figure 7F,G for HT29-5FU cells). In addition to significant inhibition of phosphorylated ERK in both cell lines (Figure 7B,D for HT29, and Figure 7F,H for HT29-5FU cells).

### 2.7. Cell Cycle Perturbation of HT29 and HT29-5FU Cells

**Figure 8:**
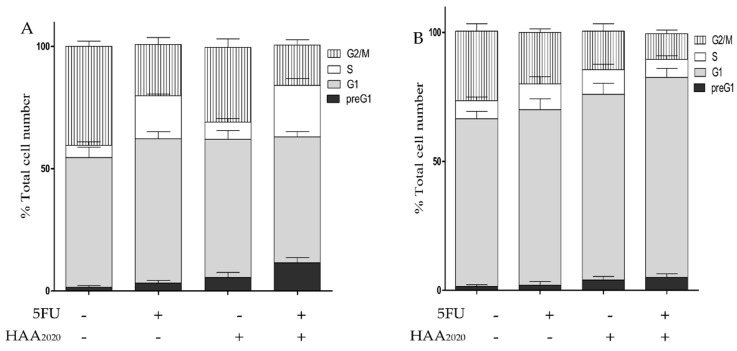
Cell cycle analysis of (**A**) HT29, and (**B**) HT29-5FU cells (mean ± SD, *n* = 3) treated for 72 h with either vehicle, 5FU (0.25 µM), HAA_2020_ (3 µM), or 5FU (0.25 µM) + HAA_2020_ (3 µM).

The perturbation of the cell cycle of HT29 and HT29-5FU cells treated with vehicle, 5FU (0.25 µM), HAA_2020_ (3 µM), and their combination are shown in Figure 8. In HT29 cells, 5FU caused increase of cells in the G_1_ and S phases compared to vehicle treatment, all at the expense of decrease or no change in the other cell cycle phases, while HAA_2020_ caused a two-fold increase in the preG_1_, and a slight increase in the G_1_ and S phases. Notably, the combination of 5FU and HAA_2020_ caused five- and four-fold increase in the preG_1_ and S cell cycle phases compared to control treatment, respectively; whereas the two compounds, either alone or combined, caused greater increase of cells in the G_1_ phase of HT29-5FU cells, compared to their effect on the HT29 cells (Figure 8B).

### 2.8. Detection of Apoptosis in HT29 and HT29-5FU Cells

**Figure 9:**
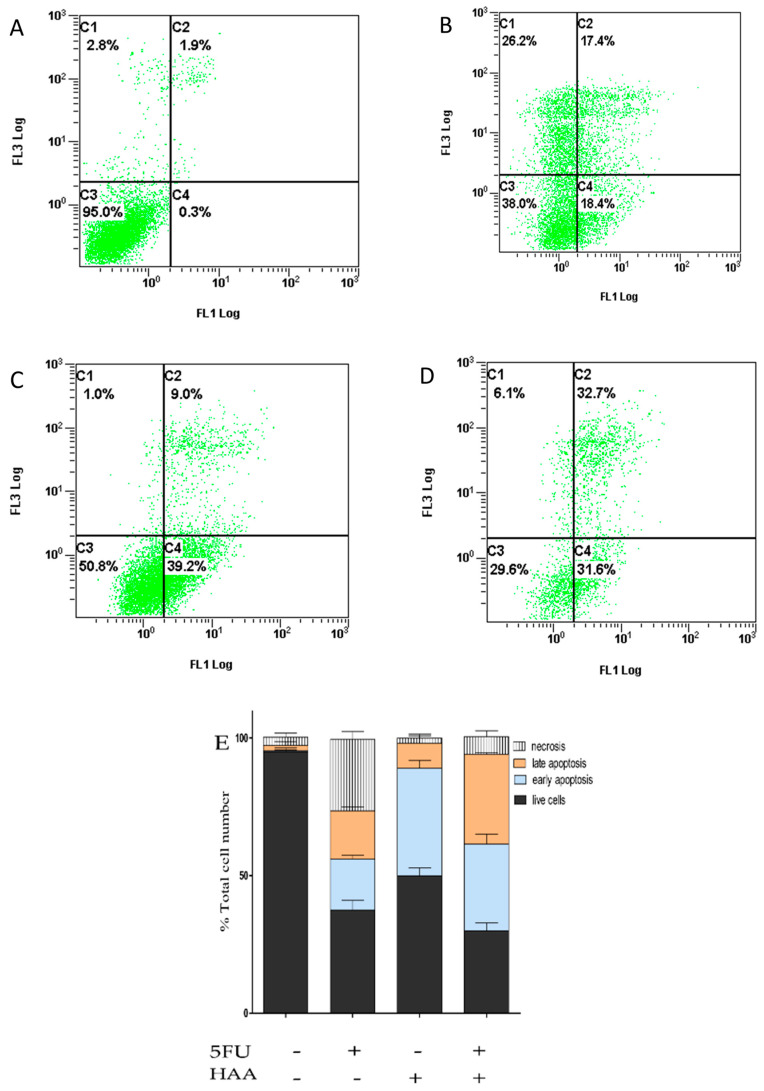

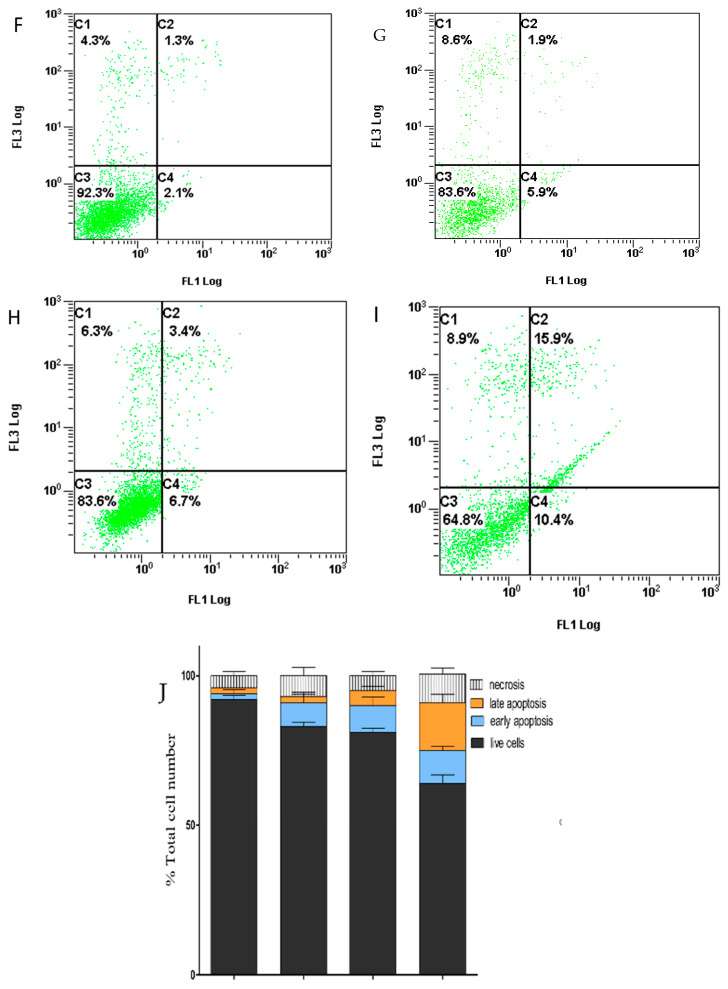
Induction of apoptosis in HT29 and HT29-5FU cells (mean ± SD, *n* = 3, x3 independent experiments), which were treated (72 h) with either (**A**,**F**) vehicle, (**B**,**G**) 5FU (0.25 µM), (**C**,**H**) HAA_2020_ (3 µM), or (**D**,**I**): their combination. Stacked histograms of the different drug treatments in (**E**): HT29 and (**J**): HT29-5FU cells. C1: necrosis, C2: late apoptosis, C3: live cells, C4: early apoptosis.

5FU, HAA_2020_, and their combination were further tested for their possible apoptosis-inducing activity in HT29 and HT29-5FU cells, which were treated with vehicle, 5FU (0.25 µM), HAA_2020_ (3 µM), and their combination for 72 h. Collected pellets were examined by Anexin V FITC/PI assay. In HT29 cells, 5FU showed 35% increase of the apoptotic events (early and late apoptosis), compared to control cells (Figure 9A–E). HAA_2020_ caused more early apoptosis (39%) compared to 5FU (18%), and to control cells (0.3%). Treating HT29 cells with HAA_2020_ and 5FU exhibited significant increase in the apoptotic events (64%). Similarly, the combination caused increase of apoptosis in HT29-5FU cells, but it was less than half of that caused in the HT29 cells (Figure 9F–J).

### 2.9. Immunofluorescence Microscopy and Western Blotting in HT29 and HT29-5FU Cells

**Figure 10:**
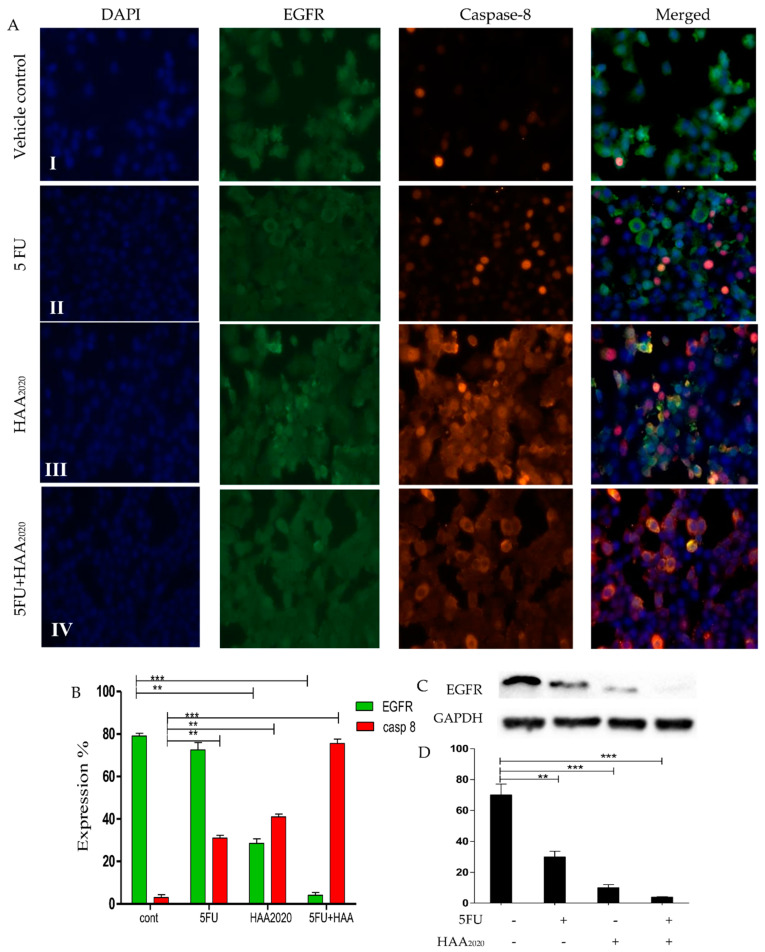

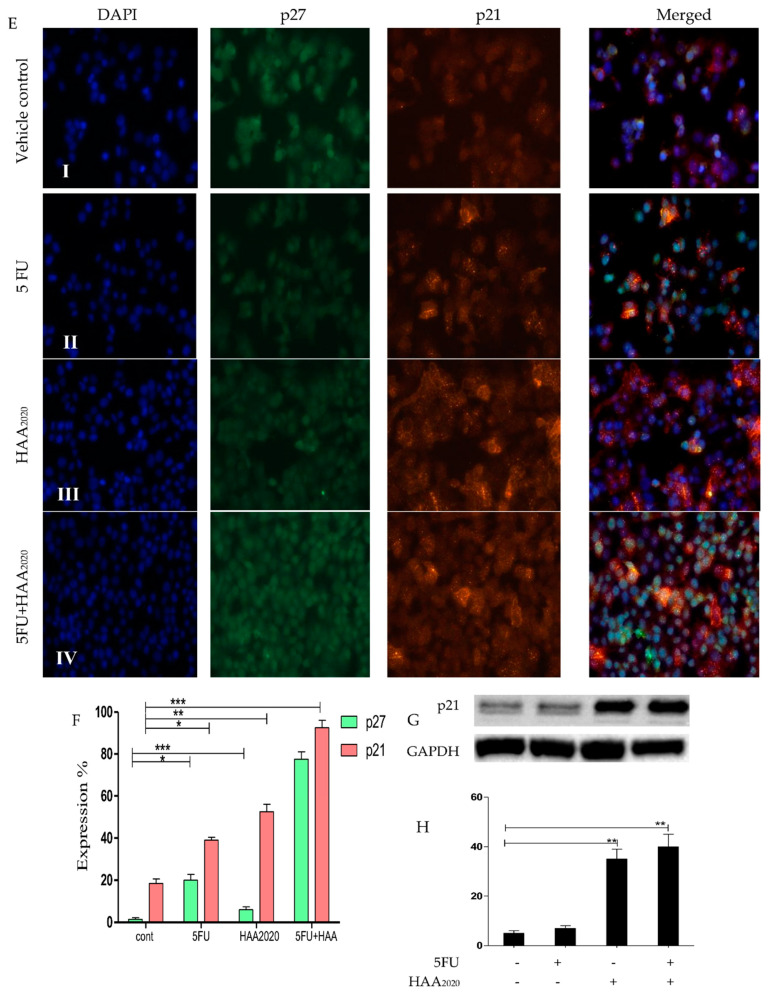
Detection of the immunofluorescence in (**A**,**B**) (expression %)**:** HT29 and (**E**,**F**) (expression %): HT29-5FU cells (EGFR and p27: green, caspase-8 and p21: red, stained with DAPI, 15 μm; 40× objective). (**I**) vehicle control, (**II**) cells incubated with 5FU, (**III**): cells incubated with HAA_2020_, (**IV**): cells incubated with 5FU and HAA_2020_ for 72 h. Quantification of EGFR level amounts in HT29 cells by Western blotting (**C**,**D**). Quantification of p21 level amounts in HT29-5FU cells by Western blotting (**G**,**H**). Results in (**D**,**H**) represented the mean ± SD of the fold change percentage (%, Y axis) of the relative protein levels normalized to GAPDH, *n* = 2, ×2 independent experiments. *p* < 0.05 (*), *p* < 0.01 (**), and *p* < 0.001 (***) were considered significant.

The combination of 5FU and HAA_2020_ showed different effects on cell cycle and apoptosis in the two cell lines. Thus, we used immunofluorescence microscopy and Western blotting to test their effects on relevant proteins. The effect on apoptosis by the combination shown by immunofluorescence microscopy was more pronounced in the HT29 cells. HAA_2020_ caused more inhibition of EGFR and greater increase of caspase 8 compared to 5FU, and their combination showed even higher effect compared to their single effects (Figure 10A,B). The effect of the combination on EGFR was confirmed by Western blotting, which showed more significant inhibition compared to the effect of each of the compounds alone (Figure 10C,D). In HT29-5FU cells, each of the two compounds and their combination caused significant increase of cells in the G_1_ phase. Consequently, and using the immunofluorescence microscopy, the combination, followed by 5FU alone caused significant increase of p27. While the combination followed by HAA_2020_ caused significant increase of p21 cell cycle inhibitor (Figure 10E,F). Western blotting of HT29-5FU cells treated with the same panel of compounds supported that result, as HAA_2020_ alone or combined with 5FU caused greater increase of p21 compared to vehicle control or cells treated with 5FU (Figure 10G,H).

## 3. Discussion

In the first part of this study, the PamChip^®^ kinase activity profiling was used for analysis of the kinases and kinase pathways in samples taken from Saudi CRC patients, which facilitated the determination and assessment of the level of kinase activities and targets; compared to other genetic sequencing methods, which are used to identify kinases of the human genome [37]. Thus, the PamChip kinase activity profiling may accelerate the drug discovery of more personalized kinase inhibitors that can overcome resistance to standard drugs. The limitation of that part of kinase pathway analysis in our study is the low number of involved patients, due to the on-surgical operational arrangements of sampling and maintenance at −80 °C, compared to the other techniques, which utilizes formalin-fixed paraffin-embedded (FFPE) samples.

The most significant of the top ten enrichment pathways was the PI3K/AKT pathway, which is important for cancer cell growth and survival. The PI3K family consists of PIK3CA, PIK3CB, PIK3CD, PIK3CG, PIK3R1, and PIK3R3, while the AKT family consists of AKT1 and AKT2. The KRAS, MAPK1, MAPK3, MAPK7, MAPK8, and MAPK14 are among the genes related to the PI3K/AKT pathway [38,39]. Three of the PI3K-related genes were detected in the Saudi CRC samples in this study: PIK3CA, BAD, and c-RAF-1 (Supplementary material S2: enrichment pathway analysis). The PIK3CA is a crucial CRC treatment biomarker, because CRC patients with PIK3CA mutation were found to be less responsive to EGFR inhibition, while their survival rates can be increased with the use of aspirin [8,40]. In a previous study, Saudi CRC patients were found to have 12% more PI3KCA mutations in comparison with counterpart CRC patients from USA and Europe [17,41]. The FDA has approved idelalisib, copanlisib, and duvelisip as PI3K inhibitors [42], and alpelisib for the PIK3CA-overexpressing CRC patients [43].

The second top enrichment pathway in this study was the inhibition of Ephrin receptors in colorectal cancer. Ephrin receptors play central roles in cell growth, differentiation, and metastasis. The biological effects of the Ephrin receptors are mediated by E-cadherin, RhoA, Rac1, and RAP-1A, which regulate repulsion, adhesion, and deadhesion mechanisms involved in motility of adherent cells. Dysregulation of the Ephrin receptors-Ephrins system is a key contributor to the progression of CRC [44,45]. Yet, the development of Ephrin inhibitors is still in its infancy, because the expression of Ephrins may be lost in advanced CRC [46]. Next, the development of the growth factors, EGFR signaling pathway, and the VEGF/VEGFR signaling were the third, sixth, and seventh top detected pathways in the Saudi CRC samples, respectively, all denoting the importance of VEGF/VEGFR and EGF/EGFR pathways which lead to angiogenesis in CRC [3,6,7]. Additionally, the PDGFRA was reported to activate PLCG1/PKC cascade, which stimulates PI3KCA, PI3KR1, PDPK1, AKT, and mTOR [47,48].

The oxidative stress ROS-mediated MAPK pathway was the top four in this study. The reactive oxygen species (ROS) has many forms including the hydrogen peroxide (H_2_O_2_), which is generated in response to many different stimuli. ROS act as second messengers and their signaling occurs via regulation of several pathways like MAPK cascades leading to ERK1/2, MAPK 7–10, and p38-MAPK activation. In particular, H_2_O_2_ directly oxidizes c-Src leading to the activation of EGFR signaling cascade (mediated by ERK1/2) and MAPK7 cascade. Furthermore, H_2_O_2_ oxidizes and activates MAP3K5, which induces MAPK8-10 and p38 MAPK cascades. Additionally, H_2_O_2_ exposure increases intracellular calcium levels, contributing to the activation of all MAPKs [49,50,51]. In the following step of our kinase pathway analysis, the network processes of the Saudi CRC samples showed the importance of the G_1_/S cell cycle phases, CDK (1, 2, 3, 5, and 14) and p21 in the development of CRC. In addition, the overexpression of the PI3K/AKT and MAPK signaling promote the anti-apoptosis processes. Moreover, the analysis of the GO process highlighted the role of kinase phosphorylation. It is evident from the kinase pathway analysis that the activation of PIK3CA/AKT, and MAPK through VEGF/VEGFR and EGF/EGFR, are the most important pathways for the development of angiogenesis, invasion, migration, and cell survival in the Saudi CRC patients. Thus, the result of the PamChip^®^ profiling agrees with the results of some previous immune-histochemistry and tissue microarray-based pathway studies [17,41].

As the drug resistance and toxicity are the main hurdles for the use or development of anticancer drugs [13], we assessed the levels of ABC transporters in the Saudi CRC samples in this study, where the ABCC1 was the highest followed by ABCG2 and ABCB1 transporters, respectively. This result agrees with a previous study which showed that the ABCC1 was the most highly expressed transporter in blood samples taken from CRC Saudi patients and disagrees in that not all of three transporters were significantly high in the samples of our study [21]. The mutated KRAS was detected in 80% of our samples. Patients No. 1 and No. 3, who had the lowest amount of the ABC transporter genes, showed no KRAS mutation. Thus, our preliminary conclusion is that the overexpression of PIK3CA could be associated with overexpression of ABCC1 and KRAS in these Saudi CRC patients.

In the next part of this study, and as we cannot add the experimental compounds to the clinical samples, we used HT29, HCT116 [52,53,54], HT29-5FU, and HCT116-5FU [23,24,25,26] CRC cells as in vitro model. We also used LY294002 as a PI3K inhibitor [55] and HAA_2020_ as a multi-tyrosine kinase inhibitor to test their possible chemosensitizing effect of the four cells to 5FU treatment. The combination of 5FU and HAA_2020_ exhibited the lowest IC_50_ and CI values among the others in HT29 and HCT116 cells, while the combinations containing LY294002 showed the highest IC_50_ and CI values. The HT29 cell line was more sensitive for the different treatments compared to HCT116 cells. HT29-5FU and HCT116-5FU cells were resistant to 5FU in this study as its IC_50_ increased 231–300-fold compared to its activity in the HT29 and HCT116 cells. LY294002 and HAA_2020_ only lost 2.42–3.5-folds compared to their activity in the parent HT29 and HCT116 cells. Only HAA_2020_ combined with 5FU showed synergistic activity in HT29-5FU and HCT116-5FU cells, accompanied with the highest fold reversal activity (FR) in the two cell lines, while again HT29-5FU cells were more sensitive to the combination compared to HCT116 cells. The clinical response and safety of 5FU in a previous clinical trial were improved by 24–74% when it was combined with oxaliplatin and irinotecan [3]. The combination of HAA_2020_ with 5FU significantly improved the selectivity of both compounds to 68.6–206 in HT29 and HCT116 cells compared to MRC5. Oppositely, the involvement of LY294002 decreased the selectivity. Testing the selectivity of all single or combined treatments in HT29-5FU or HCT116-5FU compared to MRC5 cells showed selectivity index below 1, except for HAA_2020_ or HAA_2020_ combined with 5FU, which was 1.1–2.1. Compared to HUVEC cells, the combination of HAA_2020_ with 5FU showed the best selectivity indices in the four cell lines (3–541). Thus, it was shown that while the combination of HAA_2020_ and 5FU sensitized the four CRC cells, it produced the best combination indices and resistance reversal effects in HT29 and HT29-5FU cells.

Next, the mRNA expression of the three ABC transporters in the four CRC cells without treatment showed that they were more expressed, especially ABCG2, in the HT29-5FU cells compared to the other three cell lines. HCT116 and HCT116-5FU cells showed less expression of ABCB1 and ABCC1 compared to HT29 and HT-5FU, while they did not show ABCG2. Following treatment of HT29 cells with 5FU, HAA_2020_, or their combination showed greater response of the three transporters compared with the other three cells, whereas ABCC1 was the most affected, especially the combination of 5FU with HAA_2020_. HCT116 and HCT116-5FU cells were less sensitive to treatments compared to HT29 and HT29-5FU cells. Taking together previous results, the RT-PCR was used to test the effect of 5FU, HAA_2020_, and their combination on PIK3CA, AKT, and MAPK7 expression in HT29 and HT29-5FU cells. The combination of 5FU and HAA_2020_ caused significant reduction in the expression of PIK3CA, AKT, and MAPK7 in HT29 and HT29-5FU cells. Western blotting showed significant inhibition of phosphorylated-AKT and phosphorylated-ERK in both cell lines by the combination of 5FU and HAA_2020_. In a previous report, the combination of the MAPK inhibitor (SB203580) with 5FU increased the sensitivity of HT29 and HCT116 cells to 5FU [56].

In the following step, we investigated the cell cycle perturbation of HT29 and HT29-5FU cells. 5FU caused increase of cells in the G_1_/S phase, and HAA_2020_ caused a twofold increase in the preG_1_ and a slight increase in the G_1_/S phases, while the combination caused five- and four-fold increase in the preG_1_ and S cell cycle phases, respectively. Additionally, the combination showed a pronounced increase (64%) of the HT29 apoptotic events compared to control. This induction of apoptosis was supported by increase of caspase 8 and inhibition of EGFR in HT29 cells. The combination of 5FU and HAA_2020_ pattern of effect was different in the HT29-5FU cells, as it caused more significant increase of cells in the G_1_ phase and less than apoptosis compared to its effect in HT29 cells. The p27 and p21 are important indicators for the prognosis of CRC [57,58], and their induction is associated with G_1_ cell cycle arrest and inhibition of CDKs 2, 4, and 6 [59]. Treatment of HT29-5FU cells with 5FU, HAA_2020_, and their combination showed increase of both p27 and p21, which can be associated with the G_1_ cell cycle block. Thus, combining HAA_2020_ with 5FU showed encouraging cytotoxic chemosensitizing, synergistic and selective activities in HT29, HT29-5FU, HCT116, and HCT116-5FU cells, in addition to resistance reversal activity in HT29-5FU and HCT116-5FU cells. The combination has also shown ABCC1 downregulating activity, associated with significant inhibition of PIK3CA, AKT, and MAPK7/ERK in HT29 and HT29-5FU cell lines. The combination has also shown significant increase in the preG_1_, G_1_, and S cell cycle phases associated with induction of p27 and p21, and induced apoptotic effect supported by inhibition of EGFR and increase of caspase-8 in both cells.

## 4. Materials and Methods

### 4.1. Ethical Approval, Selection of the Saudi CRC Patients, and Sampling

We received ethical approval for all protocols used in this study from the Research Advisory Council (RAC #2140006) at the King Fisal Specialist Hospital and Research Center (KFSHRC) Riyadh, Saudi Arabia, prior to starting this work. Consent was received from all study participants following the guidelines outlined in the Declaration of Helsinki. Saudi patients (*n* = 10), who were diagnosed with CRC at the KFSHRC (2016–2017), were randomly selected for this study. None of the patients were subjected to chemotherapy. Samples (~5 × ~25 μm) were collected from CRC surgery and were immediately maintained at −80 °C. RNALater was not added to the samples to retain kinase activity. Patient criteria were described in Table 1.

### 4.2. 5FU, LY294002, and HAA_2020_

Two compounds were obtained from Selleckchem (Houston, TX, USA): 5FU and LY294002. The third compound (HAA_2020_) was kindly provided by collaborators (Figure 1, [36]).

### 4.3. Maintenance of Cell Lines

HT29 and HCT116 (human colorectal adenocarcinomas, expressing MAPK-ERK, PI3K/AKT, EGFR, VEGFR, and HER2, [52,53,54]), MRC5 normal fibroblast (Medical research council-5), and HUVEC (human umbilical vein endothelial cells) were obtained from ATCC. For sub-culture of the cells, RPMI-1640 and EMEM media (1% Antibiotic-Antimycotic, 10% FBS, Gibco, Gaithersburg, MD, USA) were used under standard conditions (37 °C, 100% humidity, and 5% CO_2_). Gelatin (0.2% v/v in dH2O) was used to cover flasks before adding endothelial cells basal medium-2 (EBM-2) to sub-culture HUVEC cells under the same conditions. HT29-5FU and HCT116-5FU resistant cell lines were developed and maintained in RPMI-1640 media containing 5FU (5 µg/mL) under the same above conditions. Addition of 5FU was stopped seven days before every experiment [60].

### 4.4. Kinase Cctivity in the CRC Samples

The PamChip^®^ peptide microarray-based kinase activity profiling assays were done as previously described [32]. The immobilized peptides in the PamChip^®^ micro-arrays can be phosphorylated by the kinases present in the investigated sample(s) in the presence of ATP. Known phosphorylation sites of human proteins were used to investigate the amino acid sequences of the studied peptides. Lysis buffer was used for each of the fresh frozen colorectal tissue samples, which were then incubated for 30 min at 0 °C, spun for 15 min at 16 × 10^3^ g, and the resulting supernatants were aliquoted. Bradford assay was used for determination of the protein concentration. The activities of PTK and STK were assessed as previously described [30,31]. For quantification of the peptides’ signal on the microarrays and data analysis, the Bionaviagor 6.3 software was used (PamGene^®^ International BV, ‘s-Hertogenbosch, The Netherlands).

#### Data Interpretation

Metacore was selected for data interpretation. The study was performed based on the signal intensities of selected proteins, the enrichment pathways, networks, and GO process. The UniProt IDs were placed on canonical pathways maps based on literature. To consider a pathway as significant the *p*-value had to be >4. The blue colored-thermometers on the maps represent downregulation while the red ones represent upregulation.

### 4.5. Quantitative Real-Time PCR

The gene expression of KRAS, ABCB1, ABCC1, and ABCG2 (Table 7) was determined in the 10 Saudi patient sample lysates immediately following taking sample out of the –80 °C. The expression of ABCB1, ABCC1, ABCG2, PIK3CA, AKT, and MAPK7 was also quantified in HT29, HT29-5FU, HCT116, and HCT116-5FU cells (2 × 10^6^ cells/well), which were either not treated or treated with vehicle, 5FU (0.25 µM), HAA_2020_ (3 µM), or their combination for 72 h, by the RT-PCR (Applied Biosystems 7500 Fast Real Time PCR System) according to a previous report [61].

### 4.6. Combination Cytotoxicity and Selectivity Studies

5FU, LY294002, HAA_2020_ (0.1 µM–100 µM) and their combinations were used to treat either HT29, HT29-5FU, HCT116 and HCT116-5FU, MRC5, or HUVEC cells (3–5 × 10^3^/well) using the MTT (3-(4,5-dimethylthiazol-2-yl)-2,5-diphenyltetrazolium bromide) assay according to previous reports [62,63]. Following incubation (72 h), the MTT was added (3 h, Life technologies, Waltham, MA, USA), and PR 4100 spectrophotometer (BIORAD, Hercules, CA, USA) was used to quantify the absorbance. The IC_50_ was determined by GraphPad Prism. For calculation of the selectivity index (SI), the IC_50_ value against either MRC-5 or HUVEC cells was divided by the IC_50_ value against either HT29, HT29-5FU, HCT116, or HCT116-5FU cells. For determination of the fold reversal (FR), the IC_50_ value of 5FU against HT29-5FU or HCT116 cells was divided by the IC_50_ value of 5FU-combination against HT29-5FU or HCT116 cells. CompuSyn software was used for calculation of the combination index (CI).

### 4.7. Western Blotting

Western blotting was used to determine the expression change of ERK, p-ERK, AKT, p-AKT, EGFR, and p21. HT29 or HT29-5FU cells (0.5–1 × 10^6^ cells/well) were treated with vehicle, 5FU (0.25 µM), HAA_2020_ (3 µM), or their combination for 72 h. Lysis buffer was used to isolate the total proteins, and their concentration was determined by the Bradford method. The loading protein samples were electrophoresed on a polyacrylamide gel and transferred to a membrane. The membrane was incubated with ERK, p-ERK, AKT, p-AKT, EGFR, and p21 antibodies (Cell signaling, Boston, MA, USA) for 2 h at room temperature and the secondary antibody GAPDH for 1 h at room temperature. The immunoreactivity was visualized by chemiluminescence using horseradish peroxidase (HRP)-conjugated secondary antibodies, and their image was detected by a scanner (GeneGenome, Syngene BioImaging) [64].

### 4.8. Cell Cycle Perturbation

HT29 and HT29-5FU cells (1 × 10^6^) were stimulated separately with vehicle, 5FU (0.25 µM), HAA_2020_ (3 µM), or both compounds (72 h) following a previously reported method [65]. Ethanol (70%) was used for fixation of pellets, followed by PI staining (Santa Cruz, CA, USA). Beckman Coulter was used for flow cytometery (BC-500, Indianapolis, IN, USA).

### 4.9. Determination of Apoptosis

The possible apoptosis-inducing activity of 5FU (0.25 µM), HAA_2020_ (3 µM), and their combination (72 h) was investigated in each of HT29 and HT29-5FU cells (1 × 10^6^ cells/well) following a previously described method [66]. Following collection of the pellets and addition of the binding buffer, the Annexin V and PI (Invitrogen, Waltham, MA, USA) were added to each sample. Beckman coulter flow cytometer was used for analysis of the samples.

### 4.10. Immunofluorescence Microscopy

The immunofluorescence staining was used to test the effect of the compounds on EGFR and caspase 8 in HT29 cells, p27 and p21 in HT29-5FU cells according to a previous report [32]. Cells (2 × 10^5^/chamber) were incubated (72h) with vehicle, 5FU (0.25 µM), HAA_2020_ (3 µM), or their combination. EVOS FL microscope (40× objective) was used for slide examination.

### 4.11. Statistics

The multiple comparison tests ANOVA (one-way) with Tukey’s post hoc were used for the assessment of statistical differences.

## 5. Conclusions

The increasing interest in personalized medicines parallels the increase in the number of available drugs for treatment of cancer and the increase of drug resistance. The importance of selecting the right drug for a specific ethnicity might also be of considerable pharmaco-economic impact. By performing this study, we have highlighted some of the important targets for treatment of CRC, especially in Saudi patients. We have also tried to answer whether HAA_2020_ could have better effect on HT29, HT29-5FU, HCT116, and HCT116-5FU cells when combined with 5FU. The over-representation of PIK3CA in Saudi CRC samples in this study was associated with increased levels of ABCC1 transporter and mutated KRAS, all of which could be important determinants for choosing suitable drug combinations for Saudi CRC patients. Combining HAA_2020_ to 5FU proved promising synergistic cytotoxic and selective activities, and sensitized two types of wild and resistant CRC cells for 5FU by increasing the fold reversal activity. The combination also inhibited the ABC transporters, especially the ABCC1, downregulated PIK3CA, AKT, and MAPK7/ERK, and induced cell cycle and apoptosis in HT29, HT29-5FU cells, all which encourage further in vivo investigations of the 5FU and HAA_2020_ combination.

## Figures and Tables

**Table 1:** Colorectal cancer (CRC) patient’s criteria.

Sex	*n* ^a^	Age	*n*	BMI ^b^	*n*	CEA ^c^	*n*	No. Lymph Nodes	*n*	T-stage	*n*	LVI ^d^	*n*	KRAS	*n*
F ^e^	7	30–39	1	15–19	1	0–1	1	10–14	8	2	2	Yes	2	Yes	8
M ^f^	3	40–49	1	20–24	0	2–3	5	15–19	1	3	6	No	8	No	2
		50–59	4	25–29.9	6	4–5	1	20–24	1	4	2				
		60–69	2	≥30	3	6–7	3								
		70–79	2												

^a^, *n* = number of patients, ^b^: BMI: body mass index = weight (kg)/height m^2^. ^c^, CEA: carcinoembryonic antigen. ^d^, LIV: lympho-vascular invasion. ^e^, F: female. ^f^, M: male.

**Table 2:** Top ten enrichment pathway analysis * of the Saudi CRC samples.

Enrichment by Pathway Maps	Total	*p*-Value	FDR **	In Data	Network Objects from Active Data
PI3K/AKT pathway	50	1.303 × 10^−18^	1.521 × 10^−15^	17	BAD, NF-kB p52/p65, JAK1, NF-kB p50/p65, c-Raf-1, NF-kB p65/c-Rel, RelA (p65 NF-kB subunit), PI3K reg class IA (p85-alpha), Pyk2(FAK2), PKC-alpha, PDK (PDPK1), NF-kB, c-Src, NF-kB1 (p105), PI3K reg class IA (p85), CDK2, FAK1
Inhibition of Ephrin receptors in colorectal cancer	30	7.103 × 10^−18^	4.144 × 10^−15^	14	Ephrin-B receptors, Ephrin-A receptors, Ephrin-A receptor 2, Ephrin-B receptor 3, c-Rel (NF-kB subunit), Ephrin-B receptor 4, Ephrin-B receptor 2, Ephrin-A receptor 1, Beta-catenin, Ephrin-A receptor 3, Ephrin-B receptor 1, Ephrin-A receptor 7, Paxillin, FAK1
Development of growth factors in regulation of oligodendrocyte progenitor cell proliferation	67	1.477 × 10^−17^	5.747 × 10^−15^	18	EGFR, KV1.6, c-Raf-1, IGF-1 receptor, PDK (PDPK1), HGF receptor (Met), FGFR1, ErbB2, Vitronectin, FGFR3, PKC, PDGF-R-alpha, PI3K reg class IA (p85), Fyn, Lyn, PLC-gamma 1, PI3K reg class IA, TrkA
Oxidative stress ROS-mediated MAPK activation via canonical pathways	60	4.446 × 10^−17^	1.297 × 10^−14^	17	EGFR, ERK5 (MAPK7), CaMK II, JNK(MAPK8-10), c-Raf-1, JNK2(MAPK9), Pyk2(FAK2), CaMK II alpha, FGFR1, JAK2, SFK, CaMK II delta, c-Src, PDGF-R-beta, Fyn, PLC-gamma 1, JNK1(MAPK8)
Immune response M-CSF-receptor signaling pathway	81	5.906 × 10^−16^	1.378 × 10^−13^	18	YES, ERK5 (MAPK7), CaMK II, JAK1, c-Raf-1, M-CSF receptor, Hck, c-Cbl, Pyk2(FAK2), PDK (PDPK1), Beta-catenin, NF-kB, PLC-gamma, PKC, c-Src, PI3K reg class IA (p85), Fyn, p120GAP
Development EGFR signaling pathway	71	1.017 × 10^−15^	1.978 × 10^−13^	17	EGFR, JAK1, c-Raf-1, c-Cbl, JNK2(MAPK9), PKC-alpha, PDK (PDPK1), NF-kB, PKC-beta, JAK2, ErbB2, c-Src, PI3K reg class IA (p85), PLC-gamma 1, FAK1, JNK1(MAPK8), p120GAP
Development VEGF signaling via VEGFR2—generic cascades	93	7.941 × 10^−15^	1.323 × 10^−12^	18	NF-kB p50/p65, c-Raf-1, VEGFR-2, CREB1, Pyk2(FAK2), PKC-alpha, PDK (PDPK1), Beta-catenin, PKC-beta, Paxillin, PKC, c-Src, eNOS, Fyn, PLC-gamma 1, PI3K reg class IA, FAK1, p120GAP
Proliferative action of Gastrin in gastric cancer	53	8.453 × 10^−14^	1.233 × 10^−11^	14	EGFR, c-Raf-1, CREB1, PKC-alpha, PDK (PDPK1), Beta-catenin, PKC-beta, JAK2, PKC, c-Src, PI3K reg class IA (p85), cPKC (conventional), PLC-gamma 1, FAK1
Development: The role of GDNF ligand family/RET receptor in cell survival, growth, and proliferation	92	1.026 × 10^−13^	1.330 × 10^−11^	17	c-Raf-1, JNK2(MAPK9), CREB1, RET, CaMK II alpha, PDK (PDPK1), ATF-1, NF-kB, CREM (activators), Paxillin, VEGFR-1, c-Src, PI3K reg class IA (p85), PLC-gamma 1, CDK2, FAK1, JNK1(MAPK8)
Immune response IL-4 signaling pathway	94	1.490 × 10^−13^	1.738 × 10^−11^	17	BAD, JNK(MAPK8-10), GSK3 alpha/beta, JAK1, NF-kB p50/p65, c-Raf-1, c-Cbl, c-Rel (NF-kB subunit), CREB1, NF-kB p50/RelB, PI3K reg class IA (p85-alpha), PDK (PDPK1), PLC-gamma, JAK2, c-Fes, PKC, PLC-gamma 1

* Supplementary material S1: Enrichment pathway analysis, ** FDR: False discovery rate.

**Table 3:** IC_50_ values (72 h mean ± SD, µM), combination index and fold reversal of 5FU, LY294002, HAA_2020_, and their combinations in HT29 and HT29-5FU cells.

Drug(s) (Ratio)	HT29			HT29-5FU			
	IC_50_	CI ^a^	r ^b^	IC_50_	CI	r	FR ^c^
5FU	0.23 ± 0.04	-	0.97	68.12 ± 9.00	-	0.90	-
LY_294002_	8.67 ± 0.70	-	0.81	30.56 ± 7.31	-	0.92	-
HAA_2020_	3.75 ± 0.82	-	0.71	9.11 ± 1.99	-	0.89	-
5FU_:_ LY294002 (1:1)	0.40 ± 0.06	1.44	0.83	51.45 ± 7.31	33.12	0.93	1.3
5FU: HAA_2020_ (1:1)	0.05 ± 0.00	0.10	0.88	9.01 ± 1.33	0.80	0.88	7.5
LY_294002_:HAA_2020_ (1:1)	0.95 ± 0.09	0.31	0.95	20.05 ± 4.11	2.46	0.87	-
5FU: LY_294002_: HAA_2020_ (1:1:1)	0.09 ± 0.01	0.25	0.95	15.62 ± 2.27	12.21	0.70	4.3

^a^, CI: combination index (Fa = 0.5). ^b^, r: The linear correlation coefficient of the ME-plot, which signifies the conformity of the data with the mass-action law (an indication of how good the data are). ^c^, FR: fold reversal= IC_50_ value of 5FU against HT29-5FU or HCT116-5FU cells/IC_50_ value of 5FU-combinations against HT29-5FU or HCT116-5FU cells. Experiments were repeated ×3 (*n* = 3). (-): not applicable.

**Table 4:** IC_50_ values (72 h mean ± SD, µM), combination index and fold reversal of 5FU, LY294002, HAA_2020_, and their combinations in HCT116 and HCT116-5FU cells.

Drug(s) (Ratio)	HCT116			HCT116-5FU			
	IC_50_	CI	r	IC_50_	CI	r	FR
5FU	0.19 ± 0.03	-	0.90	44.00 ± 5.10	-	0.93	-
LY_294002_	11.54 ± 01.22	-	0.92	39.34 ± 5.12	-	0.96	-
HAA_2020_	4.11 ± 0.50	-	0.98	13.33 ± 0.65	-	0.90	-
5FU_:_ LY294002 (1:1)	3.16 ± 0.67	12.87	0.91	40.23 ± 4.10	17.70	0.92	1.1
5FU: HAA_2020_ (1:1)	0.15 ± 0.03	0.90	0.95	8.00 ± 1.12	0.95	0.90	5.5
LY_294002_:HAA_2020_ (1:1)	6.01 ± 0.89	5.11	0.89	30.88 ± 3.40	9.11	0.91	-
5FU: LY_294002_: HAA_2020_ (1:1:1)	3.76 ± 0.41	2.20	0.96	25.11 ± 3.00	17.00	0.92	1.7

**Table 5:** IC_50_ values (72 h mean ± SD, µM) and selectivity index of 5FU, LY294002, HAA_2020_, and their combinations in MRC5 cells. Experiments were repeated ×3 (*n* = 3).

Drug(s) (Ratio)	IC_50_	SI ^d^			
	MRC5	HT29	HT29-5FU	HCT116	HCT116-5FU
5FU	30.91 ± 4.22	134.4	0.4	162.6	0.7
LY_294002_	28.65 ± 2.56	3.2	0.9	2.5	0.7
HAA_2020_	19.44 ± 1.99	5.2	2.1	4.7	1.4
5FU_:_ LY294002 (1:1)	12.51 ± 1.40	31.2	0.2	3.9	0.3
5FU: HAA_2020_ (1:1)	10.30 ± 0.78	206.0	1.1	68.6	1.2
LY_294002_:HAA_2020_ (1:1)	8.79 ± 1.34	9.6	0.4	1.5	0.3
5FU: LY_294002_: HAA_2020_ (1:1:1)	5.40 ± 0.94	60.0	0.3	1.4	0.2

^d^, SI: selectivity index = IC_50_ value of a compound against either normal MRC-5 or HUVEC cells/IC_50_ value of the same compound or combination against either HT29, HT29-5FU, HCT116, or HCT116-5FU cells. Experiments were repeated ×3 (*n* = 3).

**Table 6:** IC_50_ values (72 h mean ± SD, µM) and selectivity index of 5FU, LY294002, HAA_2020_, and their combinations in HUVEC cells.

Drug(s) (ratio)	IC_50_	SI			
	HUVEC	HT29	HT29-5FU	HCT116	HCT116-5FU
5FU	11.01 ± 1.09	55.0	0.2	57.9	0.3
LY_294002_	9.02 ± 0.62	1.0	0.3	0.8	0.2
HAA_2020_	32.00 ± 3.54	8.5	3.5	7.8	2.4
5FU_:_ LY294002 (1:1)	8.56 ± 0.71	21.4	0.2	2.7	0.2
5FU: HAA_2020_ (1:1)	27.09 ± 3.00	541	3.0	180.6	3.4
LY_294002_:HAA_2020_ (1:1)	15.31 ± 2.11	16.1	0.8	2.5	0.5
5FU: LY_294002_: HAA_2020_ (1:1:1)	3.11 ± 0.19	34.5	0.2	0.8	0.1

Experiments were repeated ×3 (*n* = 3).

**Table 7:** Sequence of GAPDH, KRAS, ABCB1, ABCC1, ABCG2, PIK3CA, AKT, and MAPK7 primers.

Gene	Sequence	Gene	Sequence
GAPDH	F:AGGTCGGTGTGAACGGATTTG R:TGTAGACCATGTAGTTGAGGTCA	KRAS	F:CACTGTAATAATCCAGACTGTG R:CCCACCTATAATGGTGAATATC
ABCB1	F:TGCTCAGACAGGATGTGAGTTG R:AATTACAGCAAGCCTGGAACC	ABCC1	F:GCCAAGAAGGAGGAGACC R:AGGAAGATGCTGAGGAAGG
ABCG2	F:TATAGCTCAGATCATTGTCACAGTC R:GTTGGTCGTCAGGAAGAAGAG	PIK3CA	F:AGACACAAAACAGGCTCAGGA R:TTGAGAGAAAAACTGATAT ATTAAATGAC
AKT	F:GTGGCAAGATGTGTATGAG R:CTGGCTGAGTAGGAGAAC	MAPK7	F:ACCGAAGGACGCTTGTTAG R:AGCAGCAGCAGAACCAAT

## Data Availability

Data available on request due to ethical restrictions.

## Supplementary Materials

The following are available online. Word file S1.A: The inhibition of Ephrin receptors in colorectal cancer, S1.B: the development of growth factors, S1.C: the oxidative stress ROS-mediated MAPK activation. Excel file S2: Enrichment pathway analysis, process network, GO processes, and disease by biomarkers.

## Sample Availability

Compounds used in this study are not available from the authors.

## Abbreviations

ABC	ATP binding cassette transporters
AKT/1	V-akt murine thymoma viral oncogene/homolog 1
AKT2	V-akt murine thymoma viral oncogene homolog 2
APC	Adenomatosis polyposis coli
ATP5A1	ATP synthase subunit alpha
CTCFL	CCCTC-binding factor (zinc finger protein)-like
ERK	extracellular signal regulated kinase
FITC	fluorescein isothiocyanate
FLT-3	Fms like tyrosine kinase 3
HER2	Erb-B2 receptor tyrosine kinase 2
JAK	Janus kinase
KFSHRC	King Fisal Specialist Hospital and Research Center
KRAS	Kirsten rat sarcoma viral oncogene homolog
KIT	KIT Proto-Oncogene
MAPK	mitogen activated protein kinase
mTOR	mechanistic target of rapamycin
NARS	Asparaginyl-TRNA synthetase
NOS	nitric oxide synthase
PARP-1	Poly(ADP-Ribose) polymerase 1
PDGFRA	platelet-derived growth factor receptor alpha
PDPK1	3-phosphoinositide dependent protein kinase 1
PI	propidium iodide
PI3K	phosphoinositide-3-kinase
PIK3CA	phosphatidylinositol-4,5-bisphosphate 3-kinase regulatory subunit alpha
PIK3CB	phosphatidylinositol-4,5-bisphosphate 3-kinase regulatory subunit beta
PIK3CD	phosphatidylinositol-4,5-bisphosphate 3-kinase regulatory subunit delta
PIK3CG	phosphatidylinositol-4,5-bisphosphate 3-kinase regulatory subunit gamma
PIK3R1	phosphatidylinositol-4,5-bisphosphate 3-kinase regulatory subunit 1
PIK3R3	phosphatidylinositol-4,5-bisphosphate 3-kinase regulatory subunit 2
PKC	protein kinase C
PLC	phospholipase C
PLCG1	phospholipase C gamma 1
RAF	Raf-1 proto-oncogene
RAS	Kirsten rat sarcoma oncogene 2-delete
ROS	ROS proto-oncogene 1
SMAD4	Mothers against DPP homolog 4 (drosophila)
Src	proto-oncogene tyrosine-protein kinase
STAT	signal transducer and activator of transcription
TOP2A	DNA topoisomerase II alpha
TP53	tumor protein P53
VEGF	vascular endothelial growth factors
VEGFR	vascular endothelial growth factor receptors
EGF	epidermal growth factors
EGFR	epidermal growth factor receptors
