# Supplementary material for: Chemosensitization of HT29 and HT29-5FU Cell Lines by a Combination of a Multi-Tyrosine Kinase Inhibitor and 5FU Downregulates ABCC1 and Inhibits PIK3CA in Light of Their Importance in Saudi Colorectal Cancer
Source: Molecules. 2021 Jan 11;26(2):334. doi: 10.3390/molecules26020334 (PMC7827067; doi:10.3390/molecules26020334)
Supplement: Supplementary file 1 [file molecules-26-00334-s001.zip › molecules-1051669-SM/Supplementary S1.docx]

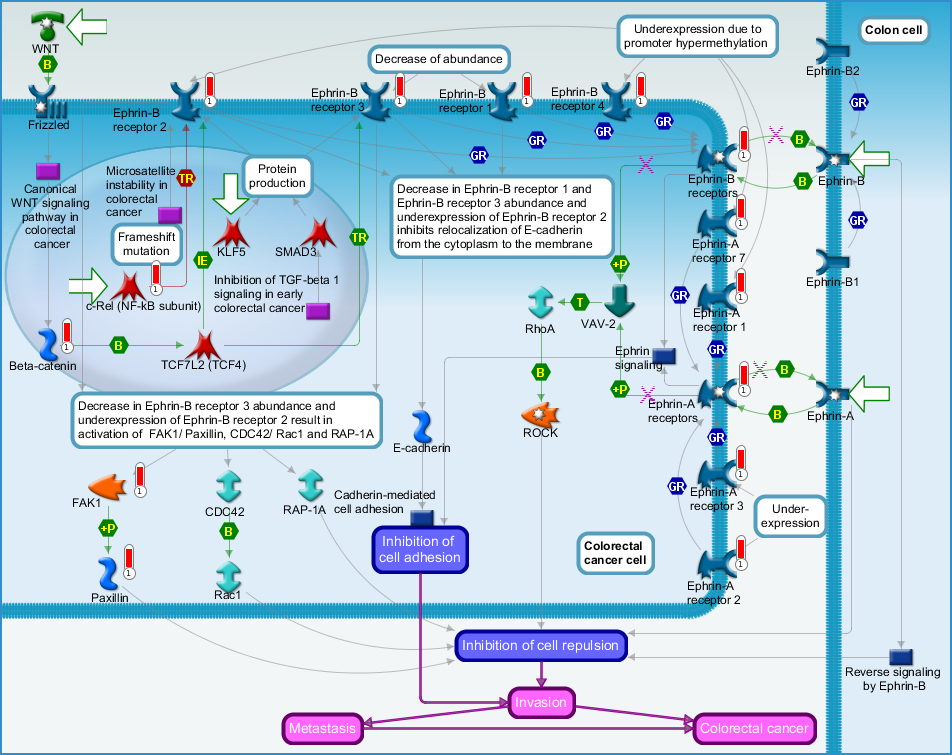


A

B


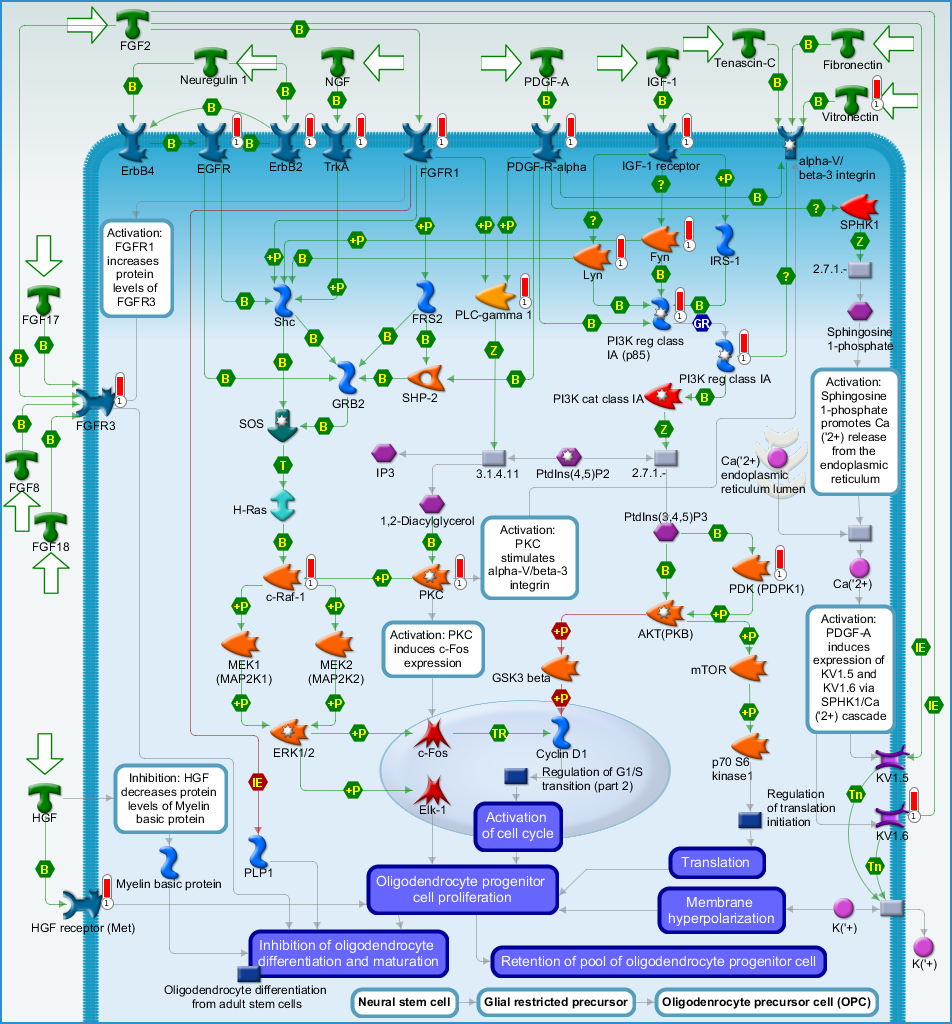


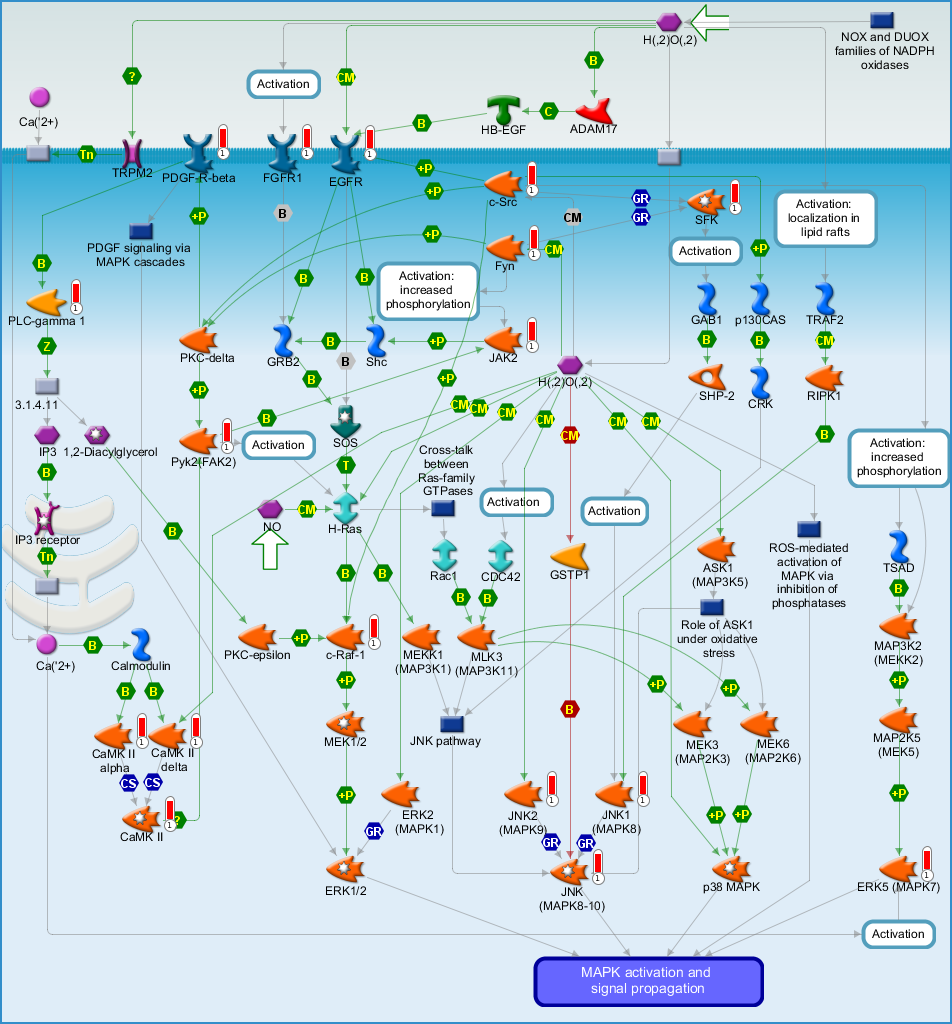


C

**Figure S1.** **A**: Inhibition of Ephrin receptors in colorectal cancer. **B:** Development of Growth factors in regulation of oligodendrocyte progenitor cell proliferation. **C**: Oxidative stress ROS‑mediated MAPK activation via canonical pathways. Experimental data are projected onto and visualized on the maps as thermometer‑like figures. Upward thermometers have red colour and indicate up‑regulated phosphorylation levels of the proteins (supplementary material S2: Key of the map).
